# Hospitalization Outcomes Among Patients With COVID-19 Undergoing Remote Monitoring

**DOI:** 10.1001/jamanetworkopen.2022.21050

**Published:** 2022-07-07

**Authors:** Bradley H. Crotty, Yilu Dong, Purushottam Laud, Ryan J. Hanson, Bradley Gershkowitz, Annie C. Penlesky, Neemit Shah, Michael Anderes, Erin Green, Karen Fickel, Siddhartha Singh, Melek M. Somai

**Affiliations:** 1 Collaborative for Healthcare Delivery Science, Center for Advancing Population Science, Medical College of Wisconsin, Milwaukee; 2 Inception Labs at Froedtert & Medical College of Wisconsin Health Network, Milwaukee; 3 Division of General Internal Medicine, Department of Medicine, Medical College of Wisconsin, Milwaukee

## Abstract

**Importance:**

Health care systems have implemented remote patient monitoring (RPM) programs to manage patients with COVID-19 at home, but the associations between participation and outcomes or resource utilization are unclear.

**Objective:**

To assess whether an RPM program for COVID-19 is associated with lower or higher likelihood of hospitalization and whether patients who are admitted present earlier or later for hospital care.

**Design, Setting, and Participants:**

This retrospective, observational, cohort study of RPM was performed at Froedtert & Medical College of Wisconsin Health Network, an academic health system in southeastern Wisconsin. Participants included patients with internal primary care physicians and a positive SARS-CoV-2 test in the ambulatory setting between March 30, 2020, and December 15, 2020. Data analysis was performed from February 15, 2021, to February 2, 2022.

**Exposures:**

Activation of RPM program.

**Main Outcomes and Measures:**

Hospitalizations within 2 to 14 days of a positive test. Inverse propensity score weighting was used to account for differences between groups. Sensitivity analyses were performed looking at usage of the RPM among patients who activated the program.

**Results:**

A total of 10 660 COVID-19–positive ambulatory patients were eligible, and 9378 (88.0%) had email or mobile numbers on file and were invited into the RPM program; the mean (SD) age was 46.9 (16.3) years and 5448 patients (58.1%) were women. Patients who activated monitoring (5364 patients [57.2%]) had a mean (SD) of 35.3 (33.0) check-ins and a mean (SD) of 1.27 (2.79) (median [IQR], 0 [0-1]) free-text comments. A total of 878 patients (16.4%) experienced at least 1 alert; 128 of 5364 activated patients (2.4%) and 158 of 4014 inactivated patients (3.9%) were hospitalized (χ^2^_1_ = 18.65; *P* < .001). In weighted regression analysis, activation of RPM was associated with a lower odds of hospitalization (odds ratio, 0.68; 95% CI, 0.54-0.86; *P* = .001) adjusted for demographics, comorbidities, and time period. Monitored patients had a longer mean (SD) time between test and hospitalization (6.67 [3.21] days vs 5.24 [3.03] days), a shorter length of stay (4.44 [4.43] days vs 7.14 [8.63] days), and less intensive care use (15 patients [0.3%] vs 44 patients [1.1%]).

**Conclusions and Relevance:**

These findings suggest that activation of an RPM program is associated with lower hospitalization, intensive care use, and length of stay among patients with COVID-19.

## Key Points

**Question:**

Is participation in a remote monitoring program for COVID-19, supported by nurses around the clock, associated with subsequent hospitalization?

**Findings:**

In this cohort study of 9378 patients, participation in a remote monitoring program was associated with lower odds of hospitalization 2 to 14 days after a positive COVID-19 test, after an adjusted analysis using inverse propensity score weighting.

**Meaning:**

These findings suggest that remote patient monitoring for COVID-19 may help patients better manage symptoms at home and help hospitals better manage bed capacity.

## Teaser

This cohort study assesses whether remote patient monitoring programs for COVID-19 are associated with the likelihood of hospitalization and whether patients who were admitted presented earlier or later for hospital care.

## Introduction

The COVID-19 pandemic has required substantial and expeditious changes to health care systems to address challenges posed toward allocating hospital resources, preventing community transmission, and treating patients.^1,2,3,4^ Particularly during the pandemic onset and the waves of more contagious variants, substantial strain has been placed on hospital systems to address shortages of staff, beds, and supplies,^5^ forcing them to implement rationing programs and measures to match patients with the most appropriate level of care.^6^ To mitigate community transmission and optimize resource utilization, health care systems have rapidly expanded telemedicine programs across all specialties.^7,8^ In addition, strategies to care for people at home using remote patient monitoring (RPM) were created to triage and manage patients with COVID-19, who may worsen during the duration of their illness.^9^ For example, patients who present with mild symptoms at initial diagnosis can deteriorate greatly during the course of their illness, requiring hospital admission and critical care.^10^ Although RPM has generally been trialed in the management of chronic disease, it may also be an effective tool to manage health care resources for more acute episodes of care.^8,11,12,13,14^

Previous studies^8,15,16^ evaluated home monitoring in patients with COVID-19 with mild symptoms during self-isolation, with early studies^17^ showing positive results with fewer admissions. Pulse oximetry at home has been successful in identifying patients needing emergent medical attention.^14,18,19,20^ It is unclear, however, whether participating in an RPM program is associated with patient outcomes or appropriate in-person resource utilization.

Our objective in this study is to evaluate the implementation of a large-scale daily RPM program for patients with COVID-19 who were managing symptoms from home. We particularly sought to assess the association of RPM with health care utilization (ie, whether RPM would be associated with increased or decreased admissions compared with those not undergoing monitoring). Secondarily, we sought to see whether patients who were eventually admitted would present earlier or later for hospital care. In these analyses, we sought to also know whether RPM was associated with inappropriate admissions (length of stay ≤1 day) or inappropriate home care (a delayed presentation leading to higher mortality, intensive care utilization, or length of stay).

## Methods

### Design, Setting, and Participants

This cohort study was conducted within an academic-community health system in southeastern Wisconsin. Froedtert & Medical College of Wisconsin Health Network provides 1.5 million ambulatory visits and cares for 55 000 hospitalized patients annually. The Health Network operates 45 clinic locations in the Milwaukee metropolitan area.

All patients who underwent COVID-19 testing at an affiliated laboratory site who tested positive were eligible for the program. Any patient could request testing either through our mobile application, patient portal, telephone, or in-person urgent care or scheduled ambulatory visits. Patients were automatically invited to enroll in the RPM program if their email address or mobile telephone number was on file at the time of testing. Patients were also invited if they had an affiliated primary care physician (PCP) within the system and had an outside positive test that was abstracted into the electronic health record (EHR). Patients signed a Health Insurance Portability and Accountability Act waiver and agreed to the program’s terms of use at enrollment. In addition to the email invitation, patients received a COVID Care Kit, a collection of educational materials, handouts, and a pulse oximeter that was available to pick up from testing sites; after October 2020, kits were automatically shipped to patients.

The program group was defined as any patient who activated the RPM program. Activation was defined as claiming an account within the RPM platform. As a sensitivity analysis, we secondarily assessed participation if the patient, in addition to activating their account, had at least 1 or more check-ins, which are programmed questions to which patients respond.

This study was reviewed and approved by the Medical College of Wisconsin institutional review board. The Strengthening the Reporting of Observational Studies in Epidemiology (STROBE) reporting guidelines for reporting observational studies were followed.^21^

### Inclusion and Exclusion Criteria

All patients were included if they tested positive for SARS-CoV-2 during the period of March 31, 2020, through December 15, 2020, at an affiliated laboratory site. Follow-up data collection for hospitalization within 14 days continued through December 31, 2020. We excluded patients younger than 18 years, those with asymptomatic tests (because these were often scheduled before procedures or other planned admissions), abstracted results, and patients who were admitted within 24 hours of a positive test. We further limited the patient sample to those who had internal PCPs to reduce the chance of missing hospitalizations.

### COVID-19 RPM Program

The COVID-19 RPM program was an automated engagement solution, supported by registered nurses, designed to improve the patient experience, coordinate care, and improve outcomes for patients with COVID-19. On March 30, 2020, the system began the program using GetWellLoop (GetWellNetwork) monitored by a centralized team of Froedtert & Medical College of Wisconsin nurses. Patients either used a responsive web application or downloaded a mobile application. Patients were asked to track their symptoms, temperatures, and pulse oximetry readings, if available. The program provided 14 days of check-ins related to progress and symptoms, in the form of questions and structured responses, while also providing a space for free-text comments (eFigure 1 in the Supplement). The program also provided educational guidance related to COVID-19, including caring for themselves at home, minimizing spread, and stress management.

A centralized virtual care team (VCT) monitored patient check-ins and free-text comments around the clock. Abnormal survey responses (eg, breathing issues or worsening fever) alerted VCT members on their dashboard. Upon reviewing the alert, the VCT contacted patients to initiate an escalation of care or to conduct further medical evaluation if warranted. VCT nurses also reacted to patient comments independently from alerts to provide education or coaching, such as how to incorporate lying in the prone position (ie, proning) into their daily care. Patients were discharged from monitoring after 18 days.

### Data Collection

Data for evaluating program outcomes were collected using reporting databases from the EHR (Epic Systems). We collected patient profile data, including sex, age, race, address, marital status, insurance, and PCP status (internal or external). Race was assessed in this study because of known racial and ethnic disparities in accessing digital health care services.^22,23,24^ We used patient addresses geocoded to the Census block level to determine the local area deprivation index, derived from the US Census and American Community Survey,^25^ as a measure of socioeconomic status and grouped by quartile.^26,27,28^

 Data about the clinical scenario (including presence of symptoms and their onset) were derived from the individual testing orders. We obtained other clinical data, including body mass index (calculated as weight in kilograms divided by height in meters squared) and comorbidities as calculated through the Charlson Comorbidity Index^29,30^ from the EHR as of the testing date. Participation in the RPM program was provided through platform usage data. These data included system registration, activation, check-ins, alerts, and comments.

### Main Outcomes and Measures

Our primary outcome was hospitalization within 14 days of a positive COVID-19 test. Hospitalizations were included if they had a flag as being related to COVID-19 through laboratory testing or hospital billing diagnosis. Hospitalizations outside the health system were not available, and readmissions were excluded from the analysis. To reduce the likelihood of missing outside hospitalizations, we limited the analysis to patients with an internal PCP because patients with external PCPs may be more likely to seek hospital care elsewhere.

To know whether RPM inadvertently may have been associated with increased mortality, such as by delaying hospitalization, we secondarily tracked patient deaths of the study cohort. Thirty-day mortality and 90-day mortality were defined as a patient marked as deceased within the EHR and/or having a death date noted within 30 or 90 days, respectively, of a positive test date. Mortality data were extracted from the EHR system in November 2021 in case death reporting in the EHR lagged.

### Statistical Analysis

Data analysis was performed from February 15, 2021, to February 2, 2022. To examine factors associated with activation, patients were divided into those who were enrolled and activated and those who were enrolled and did not activate. Age was categorized for analysis. We calculated the frequency for variables related to patient demographics and information related to personal health and COVID-19 status, along with means and SDs for time intervals, such as length of stay and time from test to hospitalization. A *t* test was used to test the mean difference of the 2 groups (RPM activated or not) in terms of the characteristics measured in continuous variables, whereas a χ^2^ test was adopted for categorical variables. For time intervals (time to hospitalization and length of stay), Wilcoxon rank-sum tests were used. Two-sided tests were used with a priori levels of significance of α = .05. All statistical analyses were performed using R programming language version 4.0.3 (R Project for Statistical Computing).^31^

To assess the association between activation and hospitalization, we performed propensity-weighted logistic regression using the glm package.^32^ With all patients being invited to use the RPM program, we used inverse propensity score weighting to account for potential bias in the observed covariates through self-selection or other mechanisms following methods by Olmos et al.^33^ Covariates included demographic variables, including age, sex, race, ethnicity, time period, insurance, marital status, Area Deprivation Index from the Census block level, encounter type where testing was ordered, Charlson Comorbidity Index score, prior hospitalization, and digital engagement proxied as having reviewed their COVID-19 test results electronically through the patient portal or the Froedtert & Medical College of Wisconsin mobile application. We also included a variable for time period (1 of the 3 waves in 2020) to allow for varying mean differences in outcomes over time due to patient behavior, including care avoidance^34^ at the beginning of the pandemic, and guideline changes during the 3 COVID-19 waves that could have affected enrollment status or clinical outcomes. The average treatment effect was calculated as the mean difference in weighted outcomes between the activated group and the invited but nonactivated group. Inverse probability weights were based on the propensity score of each patient,^35^ defined as the probability of a patient activating RPM conditional on observed covariates as described already, for doubly robust estimation.^36^

With less than 4% of the data missing observations for either socioeconomic status, race, ethnicity, or obesity, the primary analysis was run on complete cases. We used multiple imputation using the mice^37,38^ package to confirm the results. We ran additional models that included only activated RPM patients and factoring whether they engaged in the program (had check-ins digitally) or not. We also performed propensity score matching with full matching^39^ using the MatchIT^40^ package. Finally, we performed a propensity-weighted Cox proportional-hazards analysis on complete-case data using the Survival^41^ and Survminer^42^ packages. In the weighted Cox model, the starting point was the date of the test and patients were only censored at day 14.

## Results

**Figure 1.**
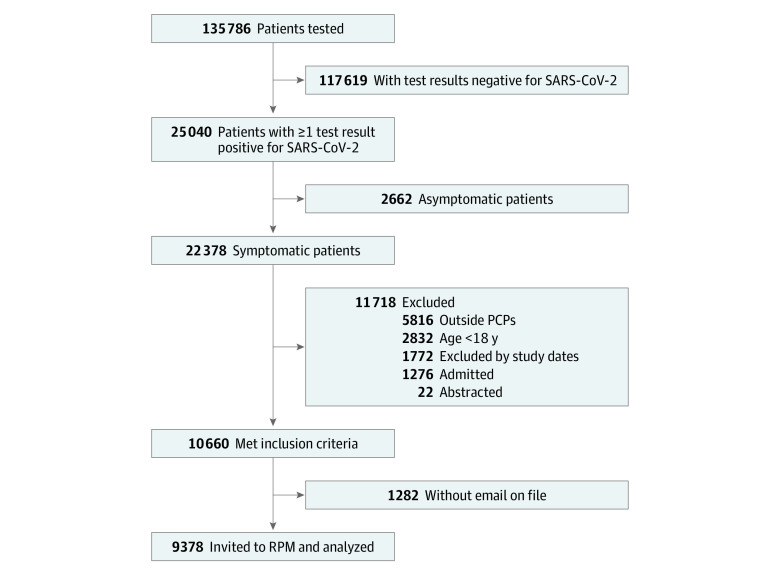
Flowchart of Study Participants PCP indicates primary care physician; RPM, remote patient monitoring.

During the study period, 135 786 patients underwent testing for COVID-19, and 25 040 patients (18.4%) had at least 1 positive test. Of these patients, 10 660 were retained for analysis after excluding those who were younger than 18 years, who did not have an internal PCP, or who were hospitalized at the time of testing (Figure 1). A total of 9378 patients (88.0%) were offered enrollment into the RPM program through an email invitation, comprising the final analytical cohort. The mean (SD) age of invited patients was 46.9 (16.3) years, and 5448 patients (58.1%) were women.

### Engagement With the RPM Program

Of the 9378 invited patients, 5364 (57.2%) activated monitoring (Table 1). Patients who activated were predominantly female (3467 of 5364 patients [64.6%] vs 1981 of 4014 patients [49.4%]). Differences in activation by race and ethnicity were not significant, although differences by comorbidities were observed. Patients with Medicare were also less likely to activate than those without Medicare (795 of 5364 patients [14.8%] vs 769 of 4014 patients [19.2%]; χ^2^_3_ = 38.65; *P* < .001). When patients requested testing through our digital platform for testing, 1805 of 2927 patients (61.7%) activated, whereas 354 of 723 patients (49.0%) with in-person visits activated (χ^2^_5_ = 61.25; *P* < .001).

**Table 1.** Demographic Characteristics of Patients Offered Remote Patient Monitoring By Activation Status

Characteristic	Patients, No. (%)		*P* value
	Activated (n = 5364)	Not activated (n = 4014)	
Sex			
Male	1897 (35.4)	2033 (50.6)	<.001
Female	3467 (64.6)	1981 (49.4)	
Age, y			
18-34	1277 (23.8)	1223 (30.5)	<.001
35-49	1663 (31.0)	1069 (26.6)	
50-64	1681 (31.3)	1005 (25.0)	
65-74	582 (10.9)	414 (10.3)	
75-89	153 (2.9)	278 (6.9)	
≥90	8 (0.1)	25 (0.6)	
Race			
American Indian or Alaska Native	17 (0.3)	11 (0.3)	.95
Asian	82 (1.5)	67 (1.7)	
Black or African American	452 (8.4)	323 (8.1)	
Native Hawaiian or other Pacific Islander	4 (0.1)	3 (0.1)	
White	4625 (86.4)	3477 (86.9)	
Other^a^	161 (3.0)	111 (2.8)	
Patient refused	12 (0.2)	7 (0.2)	
Unknown	3 (0.1)	4 (0.1)	
Ethnicity (non-Hispanic)	5143 (96.0)	3843 (96.0)	>.99
Marital status (unmarried)	3384 (63.1)	2330 (58.0)	<.001
Insurance			
Commercial	4075 (76.0)	2831 (70.5)	<.001
Medicaid	338 (6.3)	273 (6.8)	
Medicare	795 (14.8)	769 (19.2)	
Other	156 (2.9)	141 (3.5)	
Charlson Comorbidity Index comorbidities, No.			
0	3748 (69.9)	2810 (70.0)	<.001
1-2	1489 (27.8)	1023 (25.5)	
≥3	127 (2.4)	181 (4.5)	
Obesity	3156 (59.0)	2591 (64.9)	<.001
Symptoms^b^			
Shortness of breath	623 (11.6)	491 (12.2)	.38
Fever	1581 (29.5)	1146 (28.6)	.43
Cough	2984 (55.6)	2233 (55.6)	>.99
Test ordering encounter			
Electronic visit	1805 (33.7)	1122 (28.0)	<.001
In-person visit	354 (6.6)	369 (9.2)	
Portal	130 (2.4)	67 (1.7)	
Other	204 (3.8)	138 (3.4)	
Telemedicine	246 (4.6)	169 (4.2)	
Telephone	2625 (48.9)	2149 (53.5)	
Digitally engaged	5111 (95.3)	3280 (81.7)	<.001

^a^ Other denotes racial descriptions as recorded as “Other” in the electronic health record system.

^b^ Although all patients were symptomatic, only shortness of breath, fever, and cough were included in modeling.

Patients who activated had a mean (SD) of 35.3 (33.0) check-ins and a mean (SD) of 1.27 (2.79) (median [IQR], 0 [0-1]) comments with their clinical care teams. A total of 878 patients (16.4%) experienced at least 1 alert.

### Utilization and Clinical Outcomes

One hundred twenty-eight of 5364 activated patients (2.4%) and 158 of 4014 inactivated patients (3.9%) were hospitalized within 2 to 14 days of their test (χ^2^_1_ = 18.65; *P* < .001) (Table 2). The odds of admission were higher for patients with advanced age, male gender, racial and ethnic minority groups, and those with obesity or more medical comorbidities. The mean (SD) time between test and hospitalization was slightly longer among activated patients (6.67 [3.21] days vs 5.24 [3.03] days). Among those patients admitted, the mean (SD) length of stay was 2.7 days lower for RPM-activated patients than for nonactivated patients (4.44 [4.43] days vs 7.14 [8.63] days; *t* = −3.4185; *df* = 244.03; *P* = .001). Monitored patients had less intensive care use than those who were not monitored (15 patients [0.3%] vs 44 patients [1.1%]). Patients with RPM were not significantly more likely to have short (length of stay ≤1 day) hospital stays (47 of 222 patients [21.2%] vs 50 of 210 patients [23.8%]). The unadjusted 90-day mortality for all patients was 0.2% (10 patients) among activated patients and 0.6% (26 patients) among nonactivated patients.

**Table 2.** Clinical and Utilization Outcomes Among Patients by Activation Status

Outcome	Patients, No. (%)		*P* value
	Activated (n = 5364)	Not activated (n = 4014)	
Hospitalized	128 (2.4)	158 (3.9)	<.001
Length of stay, mean (SD), d	4.44 (4.43)	7.14 (8.63)	.001
Time from symptoms to hospitalization, mean (SD), d	9.84 (3.69)	8.47 (4.21)	.004
Time from positive test to hospitalization, mean (SD), d	6.67 (3.21)	5.24 (3.03)	<.001
Intensive care utilization	15 (0.3)	44 (1.1)	.001
30-d Mortality	4 (0.1)	24 (0.6)	.001
90-d Mortality	10 (0.2)	26 (0.6)	.001

### Logistic Regression With Inverse Propensity Score Weighting

**Figure 2.**
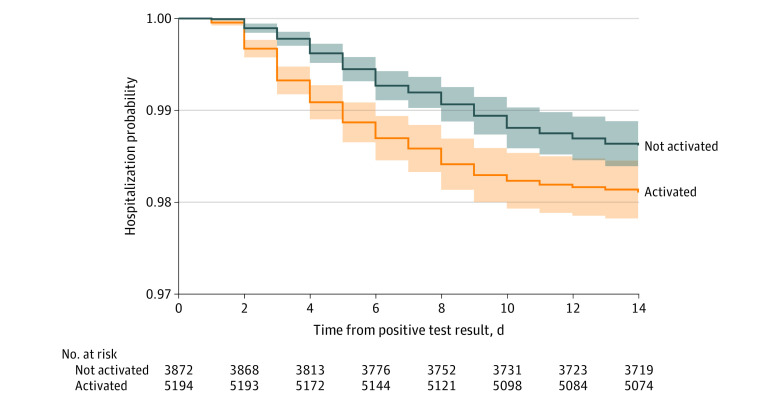
Kaplan-Meier Curve Illustrating the Inverse Probability of Hospitalization for Patients With COVID-19 Who Were Activated and Not Activated for Remote Patient Monitoring Cox proportional-hazard modeling (eTable 2 in the Supplement) was used, adjusted for age, sex, race, ethnicity, prior hospitalization, obesity, socioeconomic status, marital status, insurance coverage, time period, Charlson Comorbidity Index, and digital engagement.

In multivariable regression analysis with inverse propensity score weighting (for the likelihood of activating RPM, the C statistic was 0.67; for balance and propensity distribution, see eFigure 2 and eFigure 3 in the Supplement), activation of RPM was associated with a lower odds of hospitalization (odds ratio, 0.68; 95% CI, 0.54-0.86; *P* = .001) (Table 3). Results using multiple imputation for missing data were similar (eTable 1 in the Supplement). As a robustness check, the Cox proportional-hazard regression with inverse propensity score weighting provided similar results (Figure 2 and eTable 2 in the Supplement), as did regression with propensity score matching (eTable 3 and eFigure 4 in the Supplement). As a sensitivity analysis of only activated patients, patients with check-ins had a lower odds of hospitalization (odds ratio, 0.53; 95% CI, 0.33-0.73; *P* = .001) after weighting on propensity to have at least 1 check-in (eTable 4 in the Supplement).

**Table 3.** Risk of Hospitalization According to Logistic Regression With and Without Inverse Propensity Score Weighting

Variable	Model 1: adjusted without inverse propensity score weighting		Model 2: adjusted with inverse propensity score weighting	
	OR (95% CI)	*P* value	OR (95% CI)	*P* value
Remote patient monitoring program activation				
No	1 [Reference]	NA	1 [Reference]	NA
Yes	0.71 (0.56-0.91)	.01	0.68 (0.54-0.86)	.001
Age group, y				
18-34	1 [Reference]	NA	1 [Reference]	NA
35-49	0.95 (0.58-1.58)	.85	0.86 (0.53-1.40)	.54
50-64	2.78 (1.81-4.39)	<.001	2.59 (1.70-4.03)	<.001
65-74	3.89 (2.16-7.12)	<.001	3.68 (2.07-6.61)	<.001
75-89	4.65 (2.47-8.89)	<.001	4.11 (2.19-7.80)	<.001
90	13.29 (4.98-33.94)	<.001	14.42 (5.43-36.40)	<.001
Race				
Asian	3.14 (1.51-5.98)	.001	2.78 (1.28-5.47)	.01
Black or African	2.03 (1.35-3.01)	<.001	2.13 (1.42-3.18)	<.001
White	1 [Reference]	NA	1 [Reference]	NA
Other^a^	1.53 (0.75-2.96)	.22	1.65 (0.81-3.20)	.15
Hispanic ethnicity				
No	1 [Reference]	NA	1 [Reference]	NA
Yes	1.54 (0.74-3.06)	.23	1.57 (0.74-3.17)	.22
Sex				
Male	1 [Reference]	NA	1 [Reference]	NA
Female	0.65 (0.52-0.82)	<.001	0.65 (0.51-0.82)	<.001
Obesity				
No	1 [Reference]	NA	1 [Reference]	NA
Yes	2.2 (1.74-2.81)	<.001	2.28 (1.79-2.92)	<.001
Marital status				
Married	1 [Reference]	NA	1 [Reference]	NA
Unmarried	0.94 (0.72-1.22)	.63	0.89 (0.68-1.16)	.37
Charlson Comorbidity Index comorbidities, No.				
0	1 [Reference]	NA	1 [Reference]	NA
1-2	2.49 (1.92-3.25)	<.001	2.41 (1.86-3.15)	<.001
3	4.62 (3.18-6.68)	<.001	4.65 (3.18-6.75)	<.001
Insurance				
Commercial	1 [Reference]	NA	1 [Reference]	NA
Medicaid	1.72 (1.05-2.73)	.03	1.97 (1.23-3.08)	.00
Medicare	1.58 (1.03-2.41)	.04	1.57 (1.03-2.37)	.04
Other	1.44 (0.62-2.88)	.35	1.08 (0.41-2.31)	.87
Time period				
1	1 [Reference]	NA	1 [Reference]	NA
2	0.49 (0.31-0.83)	.01	0.51 (0.32-0.85)	.01
3	0.64 (0.39-1.09)	.09	0.71 (0.43-1.20)	.19
Area Deprivation Index quartile				
1	1 [Reference]	NA	1 [Reference]	NA
2	1.04 (0.75-1.45)	.80	1.05 (0.76-1.46)	.76
3	0.98 (0.71-1.35)	.88	0.92 (0.66-1.27)	.60
4	0.8 (0.56-1.15)	.23	0.73 (0.50-1.04)	.09
Digital engagement				
No	1 [Reference]	NA	1 [Reference]	NA
Yes	0.85 (0.64-1.14)	.27	0.96 (0.71-1.29)	.77

Abbreviations: NA, not applicable; OR, odds ratio.

^a^ Other denotes racial descriptions as recorded as “Other” in the electronic health record system.

## Discussion

In this retrospective cohort study of 9378 patients who were offered RPM for COVID-19, activation of the RPM program was associated with lower hospitalization, intensive care unit stay, and length of stay. Following an adjusted analysis using inverse propensity score weighting, patients who activated still had lower odds of being hospitalized, adjusted for observed clinical and sociodemographic characteristics.

Although a strict control group was not available, because of the rapid implementation and goal of assisting patients and clinical operations, several methods including the inverse propensity score weighting and propensity score matching were used to balance the comparison cohorts on the basis of the observed covariates. The positive outcomes seen in reducing hospitalization are similar to those described in earlier studies.^11,17,43,44,45^ We found baseline differences between patients who did and did not active RPM, similar to prior studies, although our application of the inverse propensity score weighting provides a more robust mechanism for reducing the impact of these observed differences on our result and a more advanced analytical evaluation of the association of RPM with the main study outcomes. Our study is also different because it was delivered to a more general population as well.

The mechanism underlying RPM’s association with hospitalization is not clear, but several factors may explain it. First, patients who were monitored also received education that may have affected their care trajectory, such as proning to help improve oxygenation. Second, physicians caring for patients who received the pulse oximetry and nurse support through the RPM might have felt comfortable not sending patients into the emergency department and hospital. An alternate explanation is that patients who activated were less ill, and residual confounding that could not be removed accounted for these differences. In both inverse propensity score weighting and propensity score matching, with reasonable C statistics and matching parameters, the associations persisted.

In addition to findings related to hospital admission, the data showed that patients who were admitted to the hospital while being monitored were hospitalized later than those without monitoring. Although the reasons behind such delays are not immediately known, there are different hypotheses that may explain this finding. One could be that the RPM program allowed patients to be managed and monitored at a level that would have required an admission for those who were not enrolled in the RPM—that is, either the patient or a clinician chose to defer hospital-based care because of the availability of RPM. Another factor could be that the nurses’ support through the RPM program delivered remote care that decreased risk and led to only essential admissions. This finding does not support a preintervention hypothesis that RPM would signal patients to be admitted earlier. The findings suggest that, although patients under RPM were admitted, on average, later than non-RPM patients, the late presentation was not inappropriate given that hospital length of stay, critical care utilization, and mortality were not increased in this group.

Our study also found that age and race were significantly associated with the risk of hospitalization. These results mirror the current body of evidence suggesting who is most at risk both for contracting COVID-19 and experiencing severe symptoms.^46,47,48^ To that end, although RPM and other digital interventions can be effective in deploying and allocating scarce health care resources, we must continue investigating whether such strategies exacerbate existing disparities in care utilization and outcomes. These disparities have been observed in many other digital health interventions, including video visits and telemedicine.^49,50,51^

The lessons learned from our study and others are numerous, although many questions remain regarding best practice RPM implementation for COVID-19 and other diseases and conditions. First, it remains unknown at which point in the course of disease RPM is most impactful on outcomes. Although the digital component of the RPM is easily scalable, concerns regarding other components should be carefully evaluated. In our study, we implemented a remote nurse support model. The virtual nurses had full access to the EHR and could communicate with clinical care teams. Expanding similar programs across multiple areas and diseases would require sophisticated operations and resource allocations. Further research and evaluation of these new remote operating models are warranted and should be carefully evaluated for safety.

Expansion of these programs should consider access and equity. In a previous study,^50^ we have demonstrated that access to digital technology is disproportionate among racial groups and socioeconomic statuses. Our program was accessible to anyone with a smartphone, although some technological literacy is required. Simple text messaging may also help reach additional people.

### Limitations

Some limitations of this study should be addressed. With the observational nature of the study, selection bias of healthier patients into the RPM activation exists, and although we used propensity score adjustment, residual confounding may still persist. The data were retrospective and sourced from databases, which may make the results less reliable. For example, we could not verify whether hospitalization, escalation of care, or mortality were a result of COVID-19 or another cause. To mitigate this effect, we attempted to limit follow-up data to include 15 days given the reported duration of symptoms, and we reexamined mortality data from the EHR after at least 9 months following the study period. Location and hospital system factors may have influenced enrollment rates and clinical outcomes, which could make our results less generalizable to all populations.

In addition, COVID-19 treatment guidelines (decision to place patients in the prone position or intubation recommendations), diagnosis (evolving symptom profile), and community transmission were highly dynamic during the early course of the pandemic.^52,53^ Clinical outcomes may have improved throughout the course of the pandemic. The RPM program was also rapidly updated throughout the pandemic, although the core content of monitoring, proning as tolerated, and infection control for ambulatory patients remained the same. These data were from the prevaccine era; after vaccines became available^54^ and distributed,^55^ the absolute benefit of RPM may have decreased for low-risk individuals.

## Conclusions

This report builds upon experiences from other groups showing that RPM for COVID-19 was feasible and well-received, while adding data about outcomes. Moving beyond COVID-19, these data highlight where RPM can be a helpful adjunct to care. Resource optimization is a central tenet of both RPM and pandemic-era practices. If reducing avoidable admissions can be achieved through virtual interventions, then practitioners can focus on in-person care for the most ill patients while also limiting unnecessary contact at hospitals and clinics, which is key to decreasing the spread of COVID-19. These results may also be used to establish better care guidelines for those monitoring moderate symptoms from home. Future studies should include ethnically diverse and low health care access populations, which are shown to be at higher risk for poor outcomes in COVID-19.

## Notes

Supplement. **eFigure 1.** Representative Patient Journey Through Remote Patient Monitoring Program **eFigure 2.** Plot of Standardized Mean Differences Among Covariates Before and After Propensity Weighting Used in the Main Analysis **eFigure 3.** Distribution of Propensity Scores for Activation of Remote Patient Monitoring **eTable 1.** Primary Model With Multiple Imputation Applied to Account for Missing Data **eTable 2.** Propensity-Weighted Cox Proportional Hazards Models **eTable 3.** Logistic Regression Model With Propensity Matching, Modeling Hospitalization Within 2-14 Days of a Positive COVID-19 Test **eFigure 4.** LOVE Plot Showing the Distribution of Standardized Mean Differences Between All Patients and Those Who Were Matched **eTable 4.** Sensitivity Analysis, With Propensity-Weighted Logistic Regression on Hospitalization Modeling the Use of RPM Through an Active Check-in and Covariates Among a Subset of Patients Who Activated RPM
