# Supplementary material for: Hospitalization Outcomes Among Patients With COVID-19 Undergoing Remote Monitoring
Source: JAMA Netw Open. 2022 Jul 7;5(7):e2221050. doi: 10.1001/jamanetworkopen.2022.21050 (PMC9264036; doi:10.1001/jamanetworkopen.2022.21050)
Supplement: Supplement. — eFigure 1. Representative Patient Journey Through Remote Patient Monitoring Program eFigure 2. Plot of Standardized Mean Differences Among Covariates Before and After Propensity Weighting Used in the Main Analysis eFigure 3. Distribution of Propensity Scores for Activation of Remote Patient Monitoring eTable 1. Primary Model With Multiple Imputation Applied to Account for Missing Data eTable 2. Propensity-Weighted Cox Proportional Hazards Models eTable 3. Logistic Regression Model With Propensity Matching, Modeling Hospitalization Within 2-14 Days of a Positive COVID-19 Test eFigure 4. LOVE Plot Showing the Distribution of Standardized Mean Differences Between All Patients and Those Who Were Matched eTable 4. Sensitivity Analysis, With Propensity-Weighted Logistic Regression on Hospitalization Modeling the Use of RPM Through an Active Check-in and Covariates Among a Subset of Patients Who Activated RPM [file jamanetwopen-e2221050-s001.pdf]

Supplemental Online Content

Crotty BH, Dong Y, Laud P, et al. Hospitalization outcomes among patients with COVID-19 undergoing remote monitoring. *JAMA Netw Open*. 2022;5(7):e2221050. doi:10.1001/jamanetworkopen.2022.21050

eFigure 1. Representative Patient Journey Through Remote Patient Monitoring Program
eFigure 2. Plot of Standardized Mean Differences Among Covariates Before and After Propensity Weighting Used in the Main Analysis
eFigure 3. Distribution of Propensity Scores for Activation of Remote Patient Monitoring
eTable 1. Primary Model With Multiple Imputation Applied to Account for Missing Data
eTable 2. Propensity-Weighted Cox Proportional Hazards Models
eTable 3. Logistic Regression Model With Propensity Matching, Modeling Hospitalization Within 2-14 Days of a Positive COVID-19 Test
eFigure 4. LOVE Plot Showing the Distribution of Standardized Mean Differences Between All Patients and Those Who Were Matched
eTable 4. Sensitivity Analysis, With Propensity-Weighted Logistic Regression on Hospitalization Modeling the Use of RPM Through an Active Check-in and Covariates Among a Subset of Patients Who Activated RPM

This supplemental material has been provided by the authors to give readers additional information about their work.

© 2022 Crotty BH et al. *JAMA Network Open.*

eFigure 1. Representative Patient Journey Through Remote Patient Monitoring Program. VCT: virtual care team, critical care trained nurses monitoring the RPM tool 24/7. During a patient check-in, patients answer standardized questions about their symptoms, report pulse oximeter information (where available), and consume educational content about COVID care at home, including guidance on proning and limiting spread of disease. Nurses in the VCT call patients with alerts for further intervention and triage.

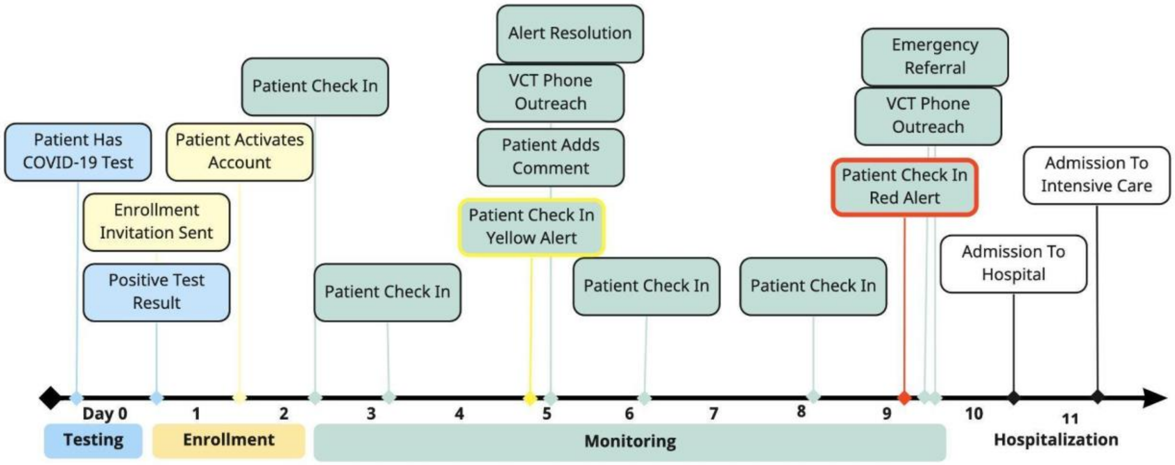

© 2022 Crotty BH et al. *JAMA Network Open.*

eFigure 2. Plot of Standardized Mean Differences Among Covariates Before and After Propensity Weighting Used in the Main Analysis. CCI: Charlson Comorbidity Index. Encounter refers to the clinical encounter where the SARS-CoV-2 test was ordered. ADI refers to area deprivation index from the American Community Survey.

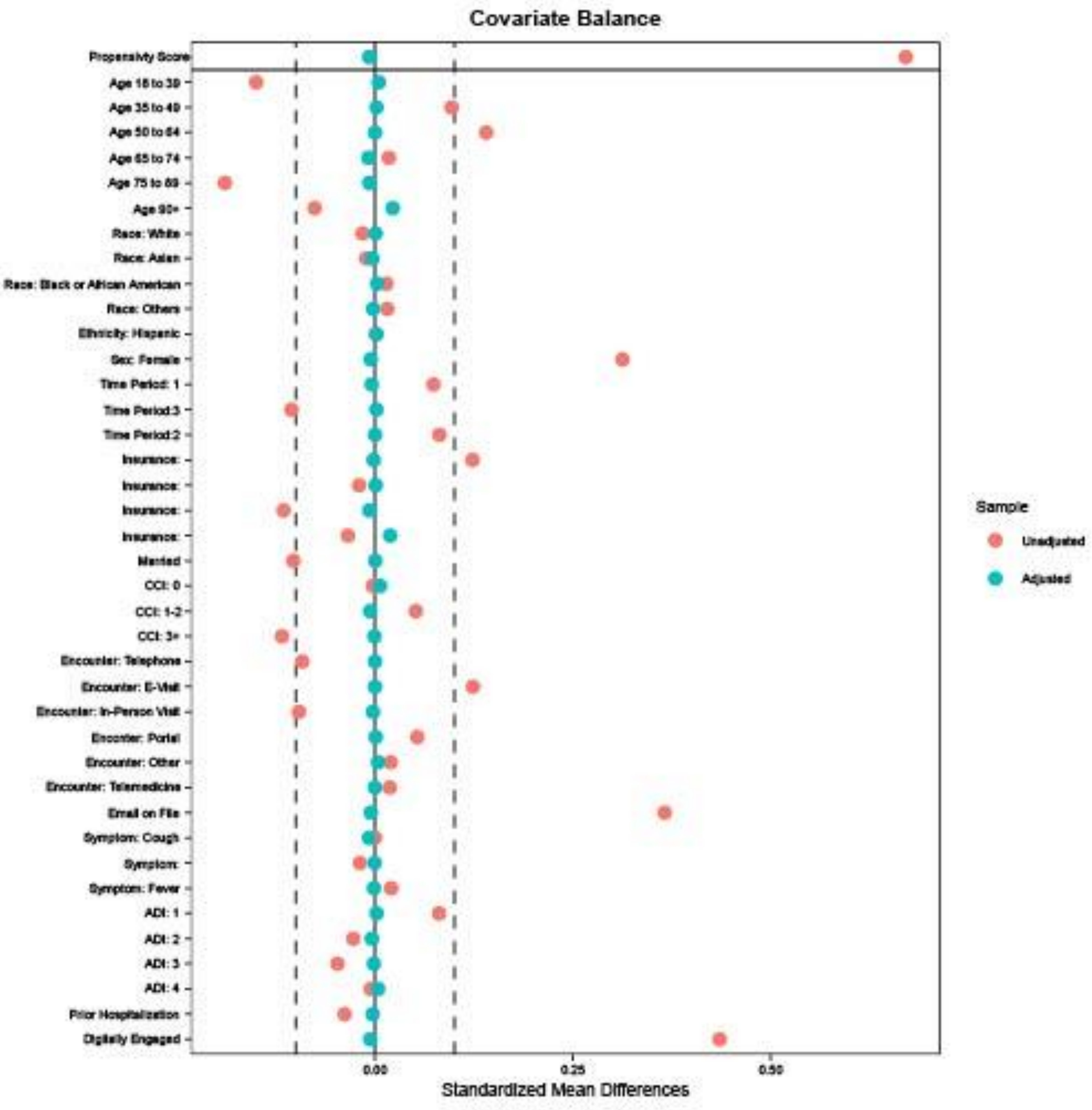

© 2022 Crotty BH et al. *JAMA Network Open.*

eFigure 3. Distribution of Propensity Scores for Activation of Remote Patient Monitoring.
Red is not activated for RPM, Teal is activated for RPM.

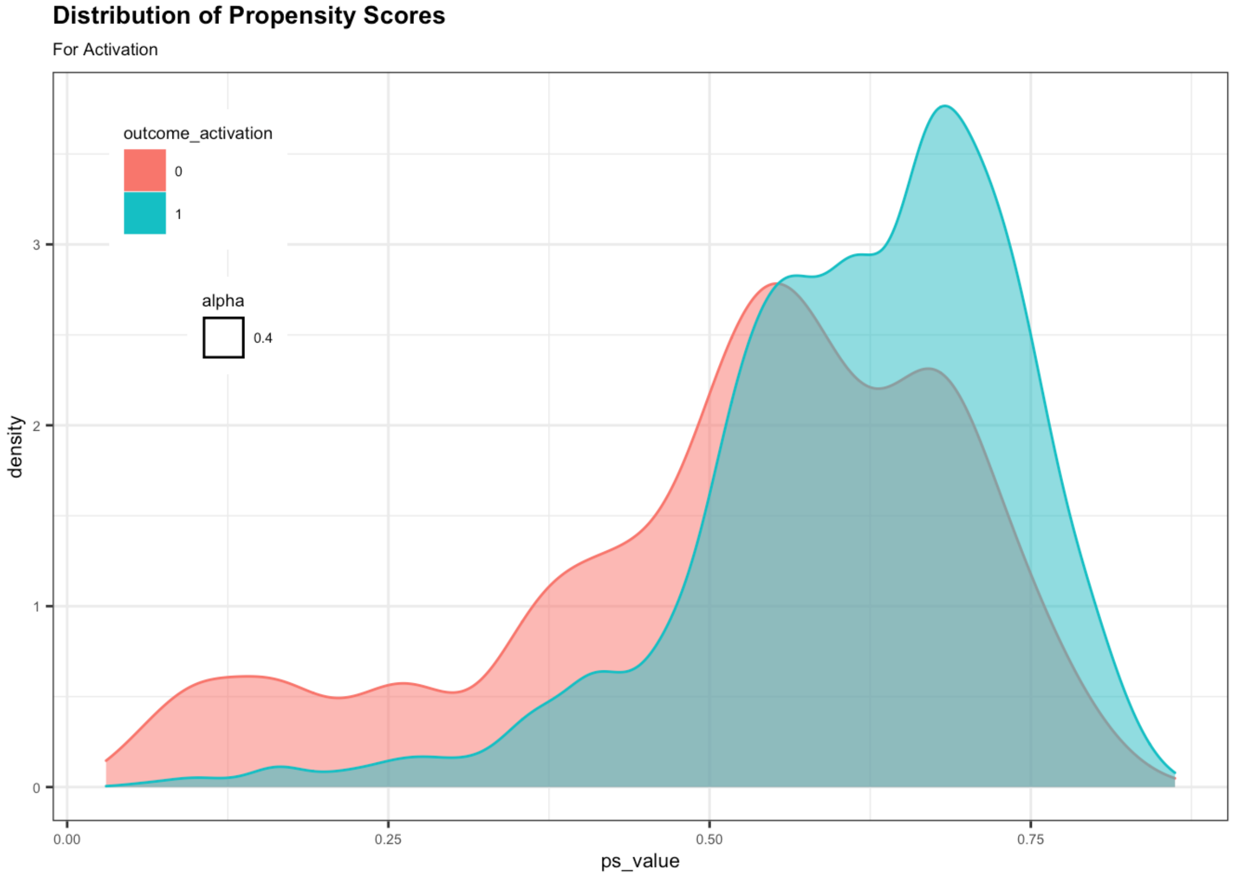

© 2022 Crotty BH et al. *JAMA Network Open.*

eTable 1. Primary Model With Multiple Imputation Applied to Account for Missing Data.

Variable	Value	OR	95% CI	p value
RPM Activation		0.71	0.56 – 0.88	**0.002**
Age	18-34			
	35-49	0.84	0.51 – 1.36	0.473
	50-64	2.55	1.67 – 3.91	**<0.001**
	65-74	3.69	2.08 – 6.54	**<0.001**
	75-89	3.9	2.08 – 7.30	**<0.001**
	90	14	5.47 – 35.80	**<0.001**
Race	White			
	Asian	3.1	1.56 – 6.16	**0.001**
	Black or African	2.07	1.39 – 3.08	**<0.001**
	Others	1.64	0.83 – 3.26	0.155
Ethnicity	Non-Hispanic			
	Hispanic	1.57	0.76 – 3.25	0.221
Gender	Male			
	Female	0.66	0.52 – 0.83	**<0.001**
Obesity	Not Obese			
	Obese	2.33	1.83 – 2.96	**<0.001**
Marital Status	Married			
	Unmarried	0.88	0.68 – 1.14	0.331
Charlson Comorbidity Index	0			
	1-2	2.49	1.92 – 3.24	**<0.001**
	3	4.75	3.28 – 6.89	**<0.001**
Insurance	Commercial			
	Medicaid	1.96	1.24 – 3.09	**0.004**
	Medicare	1.63	1.08 – 2.45	**0.02**
	Other	1.04	0.45 – 2.42	0.931
Time Period	1			
	2	0.71	0.43 – 1.17	0.176
	3	0.52	0.32 – 0.84	**0.007**
SES	1			
	2	1.05	0.76 – 1.45	0.767
	3	0.92	0.67 – 1.27	0.625
	4	0.72	0.50 – 1.04	0.082
Digital Engagement	No			
	Engaged	0.97	0.73 – 1.31	0.863

© 2022 Crotty BH et al. *JAMA Network Open.*

eTable 2. Propensity-Weighted Cox Proportional Hazards Models. Model 1 estimates the hazard ratio for RPM activation on time to hospitalization, while Model 2 estimates the hazard ratio for using RPM among the subset of patients who activated RPM. All patients were censored at 14 days.

		**Model 1: RPM Activation Among Invited**			**Model 2: RPM Use Among Activated**		
		**OR**	**95% CI**	**P value**	**OR**	**95% CI**	**P value**
RPM Activation		0.71	0.60 – 0.84	0.01			
RPM Use					0.63	0.53 – 0.75	0.001
Age	18-34	Ref					
	35-49	0.84	0.58 – 1.23	0.57	0.83	0.57 – 1.22	0.55
	50-64	2.55	1.83 – 3.55	<0.001	2.56	1.84 – 3.56	<0.001
	65-74	3.76	2.42 – 5.83	<0.001	3.71	2.39 – 5.77	<0.001
	75-89	3.8	2.34 – 6.16	<0.001	3.77	2.32 – 6.12	0.001
	90	11.25	5.72 – 22.13	<0.001	11.39	5.79 – 22.40	<0.001
Race	White	Ref					
	Asian	2.45	1.46 – 4.11	0.03	2.42	1.45 – 4.06	0.03
	Black or AA	2.08	1.54 – 2.80	0.00	2.06	1.53 – 2.78	<0.01
	Others	1.61	0.99 – 2.60	0.11	1.55	0.96 – 2.51	0.13
Ethnicity	Non-Hispanic	Ref					
	Hispanic						
Gender	Male	Ref					
	Female	0.65	0.55 – 0.78	0.00	0.64	0.54 – 0.76	0.001
Obesity	Not Obese	Ref					
	Obese	2.26	1.88 – 2.72	<0.001	2.27	1.89 – 2.73	<0.001
Marital Status	Married	Ref					
	Unmarried	0.86	0.71 – 1.05	0.33	0.86	0.71 – 1.05	0.32
CCI	0	Ref					
	1-2	2.4	1.96 – 2.93	<0.001	2.39	1.96 – 2.92	<0.001
	3	4.39	3.33 – 5.78	<0.001	4.35	3.30 – 5.74	<0.001
Insurance	Commercial	Ref					
	Medicaid	1.93	1.36 – 2.73	0.02	1.95	1.38 – 2.75	0.01
	Medicare	1.56	1.14 – 2.13	0.07	1.57	1.15 – 2.15	0.07

© 2022 Crotty BH et al. *JAMA Network Open.*

	Other	1.15	0.61 – 2.17	0.75	1.16	0.61 – 2.20	0.73
Time Period	1	Ref					
	2	0.78	0.54 – 1.13	0.48	0.79	0.54 – 1.14	0.50
	3	0.57	0.40 – 0.81	0.09	0.57	0.40 – 0.82	0.1
SES	1	Ref					
	2	1.01	0.79 – 1.28	0.97	1.01	0.80 – 1.29	0.95
	3	0.89	0.70 – 1.14	0.56	0.9	0.71 – 1.14	0.58
	4	0.72	0.55 – 0.94	0.12	0.72	0.55 – 0.94	0.13
Digital Engagement	No	Ref					
	Engaged	0.94	0.75 – 1.17	0.70	0.94	0.75 – 1.17	0.70

© 2022 Crotty BH et al. *JAMA Network Open.*

eTable 3. Logistic Regression Model With Propensity Matching, Modeling Hospitalization Within 2-14 Days of a Positive COVID-19 Test

		OR	95% CI	p value
RPM Activation		0.62	0.50 – 0.78	<0.001
Age	18-34	*Ref*		
	35-49	0.74	0.44 – 1.24	0.254
	50-64	2.9	1.90 – 4.56	<0.001
	65-74	3.32	1.84 – 6.06	<0.001
	75-89	4.08	2.15 – 7.84	<0.001
	90	16.39	6.09 – 42.00	<0.001
Race	White	*Ref*		
	Asian	3.25	1.62 – 6.04	<0.001
	Black or AA	2.15	1.45 – 3.18	<0.001
	Others	1.34	0.62 – 2.70	0.44
Ethnicity	Non-Hispanic	*Ref*		
	Hispanic	1.18	0.51 – 2.55	0.69
Gender	Male	*Ref*		
	Female	0.65	0.52 – 0.82	<0.001
Obesity	Not Obese	*Ref*		
	Obese	2.54	1.99 – 3.26	<0.001
Marital Status	Married	*Ref*		
	Unmarried	0.98	0.75 – 1.28	0.86
CCI	0	*Ref*		
	1-2	2.22	1.71 – 2.87	<0.001
	3	4.08	2.80 – 5.89	<0.001
Insurance	Commercial	*Ref*		
	Medicaid	1.79	1.09 – 2.86	0.02
	Medicare	1.58	1.04 – 2.39	0.03
	Other	0.85	0.29 – 1.93	0.73
Time Period	1	*Ref*		
	2	0.83	0.49 – 1.47	0.51
	3	0.57	0.34 – 0.99	0.04
SES	1	*Ref*		
	2	0.93	0.67 – 1.29	0.67
	3	0.83	0.60 – 1.15	0.26
	4	0.81	0.57 – 1.15	0.24
Digital Engagement	No	*Ref*		
	Engaged	0.99	0.74 – 1.34	0.93

© 2022 Crotty BH et al. *JAMA Network Open.*

eFigure 4. LOVE Plot Showing the Distribution of Standardized Mean Differences Between All Patients and Those Who Were Matched. CCI: Charlson Comorbidity Index. Encounter refers to the clinical encounter where the SARS-CoV-2 test was ordered. ADI refers to area deprivation index from the American Community Survey.

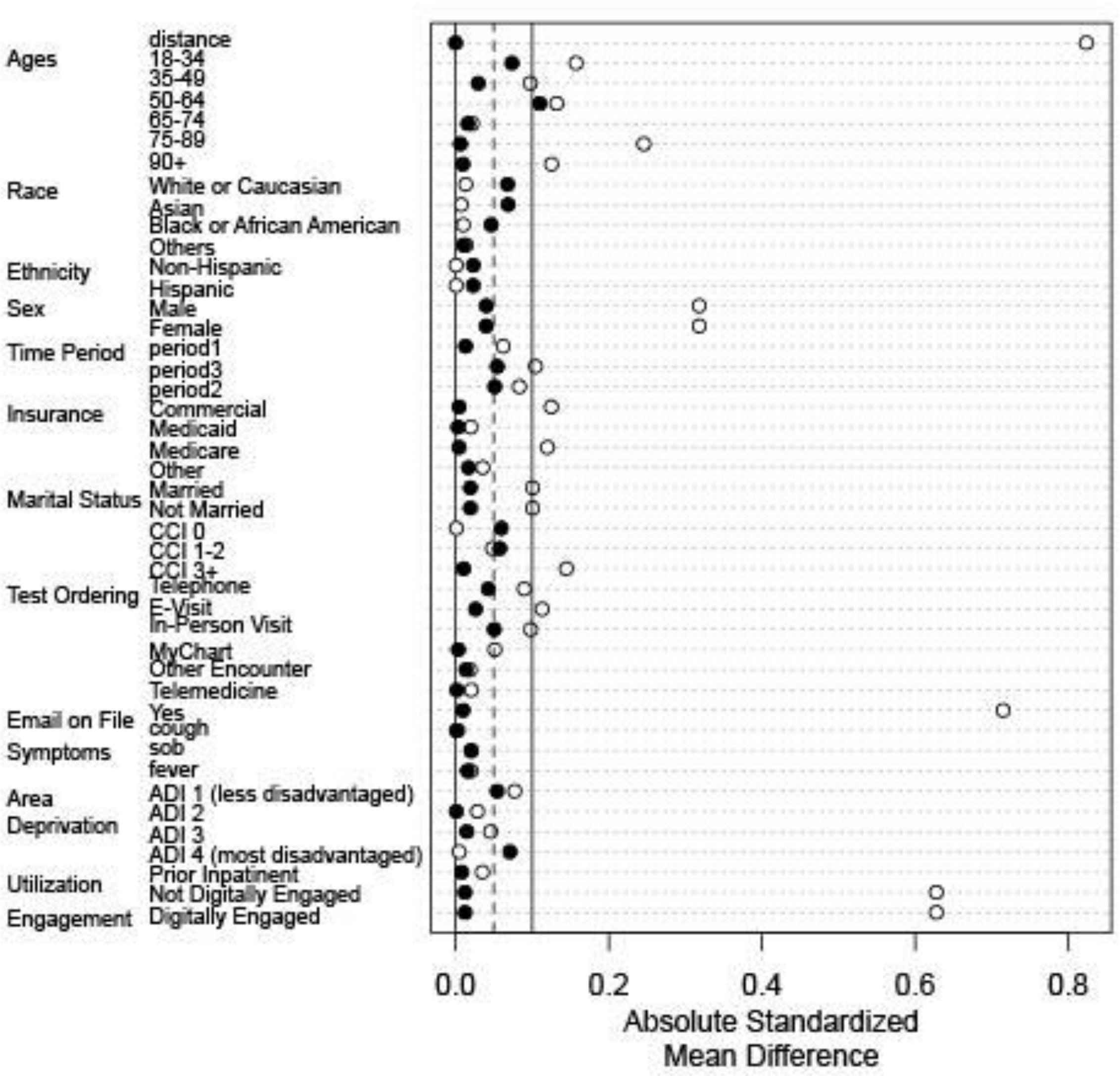

© 2022 Crotty BH et al. *JAMA Network Open.*

eTable 4. Sensitivity Analysis, With Propensity-Weighted Logistic Regression on Hospitalization Modeling the Use of RPM Through an Active Check-in and Covariates Among a Subset of Patients Who Activated RPM

**Variable**	**Value**	**OR**	**95% CI**	**p value**
RPM Use		0.50	0.33 – 0.73	**<0.001**
Age	18-34	*Ref*		
	35-49	1.71	0.62 – 5.64	0.33
	50-64	5.07	1.97 – 16.24	**0.00**
	65-74	6.93	2.11 – 26.74	**0.00**
	75-89	5.39	1.38 – 23.53	**0.02**
	90	113.5	16.13 – 790.96	**<0.001**
Race	White	*Ref*		
	Asian	2.58	0.47 – 8.94	0.19
	Black or African	1.05	0.34 – 2.82	0.92
	American	*Ref*		
	Others	0.73	0.16 – 2.52	0.64
Ethnicity	Non-Hispanic	*Ref*		
	Hispanic	1.37	0.25 – 5.23	0.68
Gender	Male	*Ref*		
	Female	0.33	0.22 – 0.48	**<0.001**
Obesity	Not Obese	*Ref*		
	Obese	4.33	2.81 – 6.86	**<0.001**
Marital Status	Married	*Ref*		
	Unmarried	0.83	0.54 – 1.30	0.40
Charlson Comorbidity Index	0	*Ref*		
	1-2	2.51	1.67 – 3.78	**<0.001**
	3	4.07	1.88 – 8.45	**<0.001**
Insurance	Commercial	*Ref*		
	Medicaid	2.81	0.94 – 7.24	**0.044**
	Medicare	2.72	1.30 – 5.59	**0.007**
	Other	4.38	1.71 – 10.04	**0.001**
Time Period	1	*Ref*		
	2	1.69	0.48 – 8.75	0.47
	3	1.33	0.40 – 6.76	0.68
SES	1	*Ref*		
	2	1.15	0.71 – 1.85	0.57
	3	0.92	0.55 – 1.53	0.75
	4	0.34	0.16 – 0.68	**0.00**
Digital Engagement	No	*Ref*		
	Engaged	2.32	0.99 – 6.59	0.08

© 2022 Crotty BH et al. *JAMA Network Open.*
